# Photorefractive keratectomy with extended ablation zone for recurrent corneal erosion syndrome accompanied with refractive errors: a study of effectiveness, safety, and refractive outcomes

**DOI:** 10.3389/fmed.2025.1592539

**Published:** 2025-07-18

**Authors:** Xinxin Yu, Chenchen Wang, Zuhui Zhang, Wuqi Zhang, Yizeng Yang, Shuangqing Wu

**Affiliations:** National Clinical Research Center for Ocular Diseases, Eye Hospital, Wenzhou Medical University, Wenzhou, Zhejiang, China

**Keywords:** recurrent corneal erosion syndrome, photorefractive keratectomy, phototherapeutic keratectomy, trans-epithelial photorefractive keratectomy, myopia

## Abstract

**Introduction:**

This retrospective study evaluated the effectiveness, safety and refractive outcomes of phototherapeutic keratectomy (PRK) with extended ablation zone in patients with recurrent corneal erosion syndrome (RCES) accompanied with refractive errors. Trans-epithelial photorefractive keratectomy (TPRK) for the contralateral eyes and phototherapeutic keratectomy (PTK) for RCES patients without refractive errors were included for comparison.

**Methods:**

The study enrolled a total of 79 eyes from 62 patients, comprising 16 eyes (16 patients) in the PRK group, 11 contralateral eyes (11 patients) in the TPRK group, and 52 eyes (49 patients) in the PTK group. The demographic and clinical profiles of the participants were meticulously documented. Surgical parameters, such as the optical zone diameter, treatment zone diameter, and ablation depth, were recorded. Postoperative duration of corneal epithelialization, recurrence of corneal epithelial erosion, complications, visual acuity and refractive error were also recorded.

**Results:**

There was no significant difference of the treatment zone diameter between the PRK group (8.92 ± 0.57 mm) and the PTK group (9.15 ± 0.48 mm), while it was significantly larger in the PTK group than that in the TPRK group (8.55 ± 0.51 mm) (*p* = 0.001). In the PRK group, recurrence of epithelial erosion occurred in one eye after PRK, which was managed conservatively. Recurrence was found in three eyes after PTK, and two eyes resolved after corneal epithelium removal followed by the application of a bandage contact lens, while one eye resolved after retreated with PTK. In terms of refractive outcomes, the deviation of target spherical equivalent at the final visit was −0.25 ± 0.57 D and −0.13 ± 0.26 D in the PRK and TPRK groups, respectively, and all patients in both groups achieved an uncorrected visual acuity of 1.0 or better. In the PTK group, 76.5%, 82.1%, and 100% of patients achieved visual acuity equal to or better than preoperative levels at 1 week, 1 month and 3 months postoperatively. The change in spherical equivalent at the last visit was +0.09 ± 0.62 D. Delayed corneal epithelial healing occurred in two eyes (12.50%) in the PRK group, one eye (9.09%) in the TPRK group and eight eyes (15.38%) in the PTK group, which correlated with the formation of no-visual interfering corneal nebula and haze. Specifically, mild corneal nebula was found in one eye in the PTK group and one eye in the PRK group due to 30 to 60 days of corneal epithelialization. Temporal haze was observed in two eyes (12.50%) in the PRK group, and two eyes (18.18%) in the TPRK group, and one eye (1.92%) in the PTK group.

**Conclusion:**

In conclusion, the effectiveness and safety of PRK with extended oblation zone were comparable with PTK for RCES and the refractive outcomes were similar with TPRK. It is recommended for RCES patients accompanied with refractive errors for relieving symptoms and acquiring encouraging visual acuity simultaneously.

## 1 Introduction

Recurrent corneal erosion syndrome (RCES) is characterized by the repeated erosion and exfoliation of the corneal epithelium, often accompanied by corneal epithelial defects (1, 2). Typical manifestations include sudden sharp pain, tears, and foreign body sensation upon waking up, which can significantly impair the quality of life of patients. Approximately 45%–64% patients have a history of epithelial trauma, such as nail or paper scratches, which is the most common reason (3, 4). Another important cause of RCES is epithelial basement (membrane dystrophy EBMD) (3, 5).

Conservative treatment for RCES primarily involves topical lubrications, anti-inflammatory agents, and corneal bandages. However, the recurrence rate remains high (6–8). For patients with refractory RCES, surgical interventions, including epithelium debridement, anterior stromal puncture, diamond burr polishing, amniotic membrane transplantation and excimer laser therapy, should be considered (8–16). Excimer laser therapies for RCES include phototherapeutic keratectomy (PTK), trans-epithelial PTK (TPTK), and photorefractive keratectomy (PRK). These surgical procedures utilize a 193 nm excimer laser to ablate the superficial corneal tissue precisely, thereby smoothing irregular corneal surfaces and enhancing epithelial adhesion (17–20). In various clinical studies, PTK has achieved success rates ranging from 60% to 100%, demonstrating its effectiveness in managing traumatic RCES, EBMD, and various hereditary stromal dystrophies (9, 21–27). In patients with RCES accompanied with refractive errors, PRK offers dual therapeutic and refractive benefits (21, 28–30). However, previous studies conducted PRK as a second step after PTK (29) or performed by zonal demarcation of PRK and PTK (28, 30), and the ablation zone for PRK were within 7 mm, whose effectiveness for RCES and results of visual acuity and refraction were various (21, 28–30). In our clinic, we performed one-step PRK with extended oblation zone for RCES accompanied with refractive errors and achieved satisfied results.

Laser *in situ* keratomileusis (LASIK) and small incision lenticule extraction (SMILE) can lead to corneal epithelial detachment during surgery and trigger postoperative RCES (31–33). Despite the absence of preoperative signs of EBMD, subclinical weakness was observed in the adherence of the corneal epithelium to Bowman’s layer. We encountered intraoperative corneal epithelial detachment during SMILE and postoperative RCES in a patient with unilateral traumatic RCES. Subsequently, trans-epithelial PRK (TPRK) became the preferred procedure for correcting refractive errors in the unaffected eyes of patients with RCES to avoid the potential risks of recurrence of corneal erosion in our clinic. For affected eyes, PRK after removal of full-corneal epithelium is recommended, to avoid the impact of irregular epithelial thickness on the correction.

This study retrospectively compared the effectiveness and safety of PRK to PTK in patients with RCES, and assessed the visual and refractive outcomes between PRK and TPRK. These findings provide a therapeutic basis for the management of RCES, particularly in patients accompanied refractive errors.

## 2 Materials and methods

### 2.1 Ethical approval

This study was conducted in accordance with the ethical principles outlined in the Declaration of Helsinki and received formal approval from the Research Ethics Committee of the Eye Hospital of Wenzhou Medical University. Ethical compliance and participant consent were rigorously maintained throughout the research process.

### 2.2 Study design

This retrospective, non-randomized, controlled study included patients with RCES who underwent either PRK or PTK at the Eye Hospital of Wenzhou Medical University between April 2021 and February 2025. All the patients in the PRK group were diagnosed with RCES accompanied with refractive errors. Meanwhile, some patients in the PRK group also underwent TPRK in their fellow eye for myopia correction. The diagnosis of RCES was established through comprehensive assessment. This included a detailed evaluation of the patient’s history of RCES-indicative symptoms, such as pain on awakening, redness, and blurred vision. Slit-lamp examination findings played a crucial role in confirming the diagnosis. Key observations included the presence of epithelial erosions, corneal microcysts, and focal epithelial defects or loose epithelium with non-uniform or negative fluorescein staining. The inclusion criteria for the PRK group were as follows. First, the patients were diagnosed with RCES. Second, the patients were consistent with the diagnosis of myopia, with a spherical equivalent ≤ −1.00 diopters (D) and suitable corneal condition. Third, these patients were willing to undergo PRK treatment. Finally, the patients were treated with PRK when the disease was quiescent or convalescent stage. The exclusion criteria for the PRK group were as follows. First, contraindications for excimer laser surgery encompassed patients with keratoconus, abnormal corneal topography, active eye infections, severe dry eye, and corneal thickness < 470 μm, etc. Second, patients with severe autoimmune diseases, such as rheumatoid arthritis, sarcoidosis, systemic lupus erythematosus, and thyroiditis, were excluded.

The demographic and clinical profiles of the participants, including their age, sex, the affected ocular laterality, the underlying etiology of RCES, and their uncorrected visual acuity (UCVA), were meticulously documented. All patients underwent detailed ophthalmic examinations prior to surgery. Surgical parameters, such as the optical zone diameter, treatment zone diameter, and ablation depth, were recorded. Postoperative duration of corneal epithelialization, recurrence of corneal epithelial erosion, complications, visual acuity and refractive error were also recorded. Delayed corneal epithelial healing was defined as the failure of complete epithelial healing 1 week postoperatively.

### 2.3 Surgical protocol

All surgical interventions were performed by two experienced corneal surgeons (W.S.Q. and D.Q.) utilizing the Schwind Amaris^®^ 1050RS excimer laser system, which is the high-performance eye laser systems for refractive and therapeutic corneal surgery. Topical 0.5% proparacaine was administered 2–5 min before surgery. For PTK, the entire corneal epithelial layer was mechanically removed using a hockey knife. The excimer laser was then employed to ablate the Bowman’s layer and superficial stromal layer with an ablation depth of 9–39 μm. The diameter of the treatment zone, including transition zone, ranged from 8.50 to 10.00 mm. Deeper ablation was performed in patients with potholes intraoperatively secondary to multiple episodes of recurrent erosion. For PRK, the entire corneal epithelium was removed, as for PTK. The excimer laser ablated the Bowman’s layer and anterior stromal tissue with an ablation depth of 18–113 μm and the diameter of the treatment zone was 7.86–9.62 mm. For TPRK, a standardized epithelial profile with a central thickness of 55 μm was applied. The ablation depth was 43–117 μm, while the diameter of the treatment zone was 8.01–9.53 mm. In terms of surgical design, presbyopia was taken into consideration, and for the non-dominant eye, a certain degree of myopia, ranging from - 0.50 to -1.50 D, was retained. The mean target residual diopters of the PRK and TPRK groups were −0.17 ± 0.45 D and −0.43 ± 0.62 D, respectively. Postoperatively, one drop of tobramycin-dexamethasone eye drops was instilled into the conjunctival sac and a bandage contact lens was applied to the operating eye to protect and promote healing. All treated eyes were prescribed a regimen consisting of ofloxacin eye drops, artificial tears, and 0.1% fluorometholone eye drops, each administered four times daily. We recommend discontinuation of levofloxacin eye drops after removal of the bandage contact lens and maintenance of artificial tears for 6 months postoperatively. For the first month, 0.1% fluorometholone eye drops were administered four times daily, followed by a monthly tapering regimen over the subsequent 4 months. However, in cases of corneal haze development, the duration and frequency of fluorometholone treatment should be appropriately extended and increased, with careful monitoring for potential steroid-related complications, such as elevated intraocular pressure or cataract formation.

### 2.4 Follow-up

Routine postoperative follow-up appointments were scheduled at 3 days, 1 week, 1, 3, 6 months, and annually thereafter to monitor recovery and outcomes. If complete epithelialization was not achieved at 1 week postoperatively, visits were more frequent. The bandage contact lenses were removed after the epithelium healed. The patients were monitored more frequently in the event of discomfort or unforeseen issues.

### 2.5 Statistical analysis

Statistical analyses were conducted using IBM SPSS Statistics for Windows, version 26.0. To assess the distribution of the datasets, the Kolmogorov–Smirnov test was used to evaluate normality. Descriptive statistics, such as the mean ± standard deviation or median (interquartile range), were applied to summarize the key characteristics of the data. Additionally, *Pearson* or *Spearman* ’s correlation were used to analyze the correlation. Analysis of variance was used to compare the differences in surgical parameters between different groups. A *p*-value < 0.05 was established to determine statistical significance.

## 3 Results

The study included 79 eyes (62 patients), including unilateral eyes in 93.5% of cases, with an equal distribution between the left and right eyes (29 eyes). Bilateral presentation was observed in four patients, accounting for 6.5% of the cases. The mean age of the patients was 45.9 ± 10.6 years (range, 29–66 years). Of the 62 patients diagnosed with RCES, 35 were male and 27 were female. A history of trauma preceded RCES in 31 eyes (45.6%); EBMD was present in 17 eyes (25.0%); and 20 eyes (29.4%) were idiopathic (no evidence of dystrophy or trauma). Notably, two eyes developed RCES following the SMILE and LASIK procedures, which were classified as traumatic factors. Fifty-two eyes in 49 patients underwent PTK, 16 eyes in 16 patients underwent PRK, and 11 eyes in 11 patients underwent TPRK. Three eyes of two patients underwent a second PTK procedure because of recurrence after local area PTK; however, only the results of the second surgery were included in the analysis. Table 1 presents the basic characteristics of the patients with RCES in the three groups. Patients in the PRK and TPRK groups were significantly younger than those in the PTK group (*p* < 0.05). The mean post-operative follow-up was 6.9 ± 9.1 months (range: 0.3–43.0 months).

**TABLE 1:** Basic characteristics of patients in the three excimer laser treated groups.

Variables	PTK group (*N* = 49)	PRK group (*N* = 16)	TPRK group (*N* = 11)
Age (y), mean ± SD	47.6 ± 10.7	40.5 ± 7.6	40.4 ± 8.3
Gender (male/female)	25/24	12/4	9/2
Laterality (left/right/both)	21/24/4	8/7/1	5/6/0
Etiology of recurrent erosions (trauma/dystrophy/idiopathic)	23/10/16	8/4/4	6/1/4

### 3.1 Comparison of surgical parameters

This study compared the differences in surgical parameters between the different treatments, as shown in Table 2. The optical zone diameter was significantly larger in the PTK group (8.61 ± 0.50 mm) and in the PRK (7.73 ± 0.68 mm) than the TPRK group (7.24 ± 0.49 mm) (*p* < 0.001). No significant difference was observed in the treatment zone diameter between the PRK group (8.92 ± 0.57 mm) and the PTK group (9.15 ± 0.48 mm) (*p* > 0.05). However, the PTK group exhibited a significantly larger ablation zone compared to the TPRK group (8.55 ± 0.51 mm, *p* = 0.001). The ablation depth of the stroma was significantly greater in the PRK group (63.68 ± 24.43 μm) and TPRK group (72.09 ± 23.73 μm) than in the PTK group (17.45 ± 6.57 μm) (*p* < 0.001).

**TABLE 2:** Surgical parameters in the three excimer laser treated groups.

Parameters (eyes)	PTK group (*N* = 52)	PRK group (*N* = 16)	TPRK group (*N* = 11)	*P* ^1^	*P* ^2^	*P* ^3^
Optical zone diameter (mm)	8.61 ± 0.50	7.73 ± 0.68	7.24 ± 0.49	< 0.001^a^	< 0.001^a^	0.023^a^
Treatment zone diameter (mm)	9.15 ± 0.48	8.92 ± 0.57	8.55 ± 0.51	0.123	0.001^a^	0.073
Ablation depth of the stroma (μm)	17.45 ± 6.57	63.68 ± 24.43	72.09 ± 23.73	< 0.001^a^	< 0.001^a^	0.300

^1^PTK group vs. PRK group. ^2^PTK group vs. TPRK group. ^3^PRK group vs. TPRK group. ^a^*p* < 0.05. PTK, phototherapeutic keratectomy; PRK, photorefractive keratectomy; TPRK, trans-epithelial photorefractive keratectomy.

### 3.2 Changes in visual acuity and spherical equivalent

**FIGURE 1:**
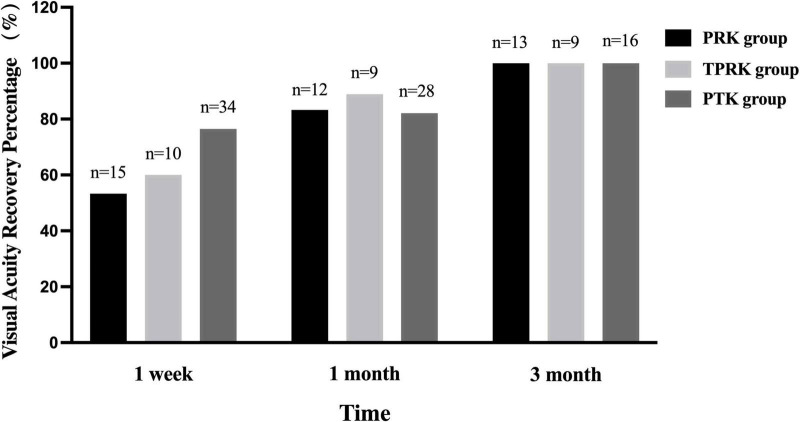
The visual acuity recovery percentage across the three groups. The percentage of PRK and TPRK achieving a uncorrected visual acuity as 1.0 or better increased gradually within 3 months postoperatively, so as the percentage of PTK recovering preoperative visual acuity. PTK, phototherapeutic keratectomy; PRK, photorefractive keratectomy; TPRK, trans-epithelial photorefractive keratectomy.

**FIGURE 2:**
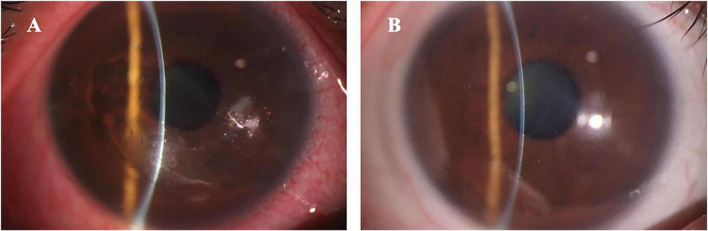
The anterior segment photography in a typical patient with recurrent corneal erosion syndrome accompanied with refractive errors. **(A)** Shows the preoperative loosing corneal epithelium with inflammation (uncorrected visual acuity was 0.1 and spherical equivalent was –5.50 D); **(B)** Shows the postoperative smoothing cornea without inflammation (uncorrected visual acuity was 1.0 and spherical equivalent was –0.37 D).

Before surgery, the spherical equivalent in the PRK and TPRK groups were −3.57 ± 1.61 and −4.43 ± 1.40 D. At the last visit, all eyes had achieved a UCVA of 1.0 or better in both groups. The mean deviation of targeted spherical equivalent was −0.25 ± 0.57 D in the PRK group and −0.13 ± 0.26 D in the TPRK group. In the PRK group, at 1 week, 1 and 3 months postoperatively, 53.3%, 83.3%, and 100% of the patients achieved a UCVA of 1.0 or better than the preoperative levels, respectively (Figure 1). Patient satisfaction with excimer laser treatment outcomes was high, as illustrated by a representative case in Figure 2. In the TPRK group, 60.0%, 88.9%, and 100% of the patients achieved a UCVA of 1.0 or better than preoperative levels at 1 week, 1 and 3 months postoperatively, respectively (Figure 1). Using the available data, in the PTK group, at 1 week, 1 and 3 months postoperatively, 76.5%, 82.1%, and 100% of the patients achieved visual acuity equal to or better than the preoperative levels, respectively (Figure 1). The mean change in the spherical equivalent between pre-operation and the last visit was + 0.09 ± 0.62 D in the PTK group.

### 3.3 Efficacy and safety

**FIGURE 3:**
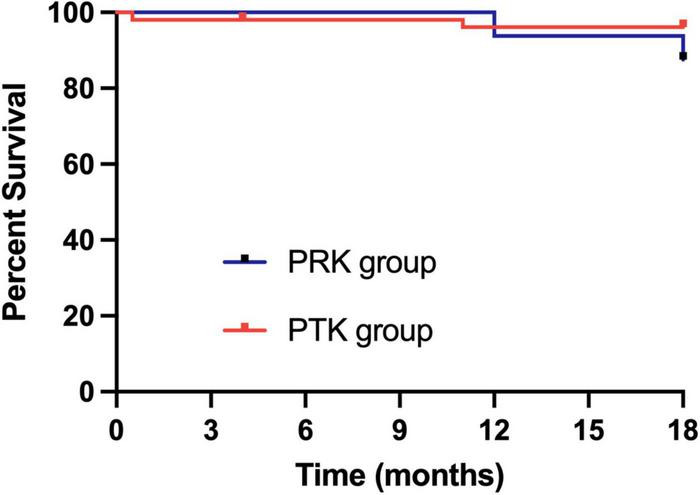
Kaplan–Meier plot demonstrating recurrence-free survival following PRK and PTK treatment. PRK, photorefractive keratectomy; PTK, phototherapeutic keratectomy.

The Table 3 showed complications and recurrences in the three excimer laser treated groups. Recurrence of epithelial erosion occurred in one eye (6.25%) at 1 year postoperatively in the PRK group and was treated conservatively. Recurrence occurred in three eyes (5.77%) in the PTK group. Among them, two eyes underwent corneal epithelium debridement combined with bandage contact lens application, while one eye underwent repeat PTK at 1 year postoperatively. Although there were no statistically significant differences in the recurrence rates between the two groups, the PRK cohort demonstrated milder clinical manifestations during recurrence episodes. Correlation analysis revealed no significant association among the optical zone diameter, treatment zone diameter, ablation depth and surgical success rate, which refers to the absence of corneal epithelial erosion recurrence during the follow-up period. The Kaplan–Meier plot demonstrated recurrence-free survival following PRK and PTK treatments (*p* = 0.22) (Figure 3).

**TABLE 3:** Complications and recurrences in the three excimer laser treated groups.

Complications (eyes)	PTK group (*n* = 52)	PRK group (*n* = 16)	TPRK group *(n* = 11)
Delayed corneal epithelial healing (*n*/%)	8 (5.38%)	2 (12.50%)	1 (9.09%)
Haze (*n*/%)	1 (1.92%)	2 (12.50%)	2 (18.18%)
Corneal nebula (*n*/%)	1 (1.92%)	1 (6.25%)	0
Recurrence (*n*/%)	3 (5.77%)	1 (6.25%)	0

PTK, phototherapeutic keratectomy; PRK, photorefractive keratectomy; TPRK, trans-epithelial photorefractive keratectomy.

Delayed corneal epithelial healing occurred in eight eyes (15.38%) in the PTK group. Among them, two eyes underwent epithelialization 11 days postoperatively. The other six eyes underwent scarping of abnormal newly-formed epithelium at 2–3 weeks and five eyes finished re-epithelialization in the following 1–2 weeks, and only one eye underwent re-epithelialization at 60 days after surgery. The mean duration of complete epithelialization after PTK was 6.42 ± 9.34 days (range 3–60 days). In the PRK group, delayed epithelial healing occurred in two eyes (12.50%), and one eye finished epithelialization in 2 weeks with conservative treatment; the other eye had abnormal new epithelium scraped at 2 weeks and finished epithelialization at 30 days postoperatively. The mean duration of epithelialization of PRK was 7.40 ± 6.93 days (range 3–30 days). In the TPRK group, all eyes finished epithelialization within 1 week, except one eye, which finished at 2 weeks postoperatively, with a mean duration of epithelialization of 5.45 ± 3.44 days (range 3–14 days).

Delayed corneal epithelial healing may result in temporary haze and a mild corneal nebula. One eye with PTK and one eye with PRK showed mild corneal nebula, whose epithelialization was completed 60 and 30 days after surgery, respectively. Haze formation was found in one eye, two eyes and two eyes in the PTK, PRK, and TPRK groups, respectively, which were not visually significant and resolved within 3–6 months. Corneal haze was observed in two eyes in the TPRK group, both of which belonged to the same individuals in the PRK group.

## 4 Discussion

Recurrent corneal erosion syndrome was first described by Hansen et al. in 1872 and has remained a clinically challenging condition for over a century. The histopathology of RCES relieved abnormalities in hemidesmosome formation and function, along with focal absence of the basement membrane in affected patients. These abnormalities result in a loose connection between the corneal epithelium and Bowman layer (1–3). To address RCES, partial ablation of Bowman’s layer and stroma using an excimer laser was employed. This procedure enhances the adhesion of basal epithelial cells to the stroma and remove debris that may interfere with proper epithelial cell function, thereby reducing the recurrence of RCES (18, 19, 34).

The efficacy of excimer lasers in RCES has been extensively validated in numerous studies; however, the results vary with the recurrence rates ranging from 0% to 40% (9, 21, 24). At the final follow-up visit, primary recurrence rates of 6.25% and 5.77% were observed in the PRK and PTK groups in our cohort, respectively. The diverse etiology of RCES may be play a role in prognosis and the success rate seemed to be higher in traumatic RCES cases compared to those with EBMD (26, 34). In addition, surgery related factors can influence prognosis, including the removal of corneal epithelium prior to laser oblation, the methods employed for epithelium removal, the design of the treatment area and ablation depth, and any additional interventions. Variations in surgical equipment and energy settings may also have contributed to the observed differences in treatment outcomes.

Early approaches to PTK involved localized laser treatment after removal of the epithelium in the affected area. These studies have reported success rates of 76.5% and 83.0%, respectively (27, 35). The current protocols predominantly employ complete limbal-to-limbal epithelial removal (9, 30, 34). Pre-laser epithelial debridement methods include manual scraping (19, 22, 36, 37) and alcohol-assisted removal (11, 23, 24, 38). In TPTK mode, the epithelium is directly removed using an excimer laser (17, 36, 39, 40). Two studies have suggested that the therapeutic effect of TPTK is superior to that of PTK with manual scraping of the epithelium (17, 36). While TPTK can remove epithelium with a smoother surface, we speculate that the uniform setting of epithelial thickness may overlook the irregular corneal epithelial thickness observed in RCES, making it potentially more suitable for the quiescent phase of RCES. We removed the epithelium from limbs to limbs by manual scraping to perform laser treatment with extended ablation zone. This approach eliminates the interference from variations in epithelial thickness and yields more predictable results. This is especially important for PRK treatment in patients with RCES accompanied refractive errors, as scraping the corneal epithelium with irregular thickness can improve refractive predictability and reduce astigmatism likelihood.

In terms of treatment area design and oblation mode, early protocols focused on lesion-centric ablation with overlapping laser spots (27, 35) or central 6–7 mm ablation combined with 3–4 mm spot PTK overlapping ablation at the peripheral area (30, 41, 42). At present, the treatment area usually crosses over the pupil with a diameter of 7–10 mm, along with the full corneal removal of the epithelium, as recommend. In our cohort, two cases (three eyes) treated with locally trapezoidal designs in the first PTK (data not shown), experienced postoperative recurrence that resolved after secondary PTK or PRK with an extended ablation zone. This suggests that patients with bilateral disease may have basal membrane defects and that local treatment cannot prevent recurrence in untreated areas. Additionally, irregularities at the junction of the laser treatment may predispose patients to recurrence.

Regarding the relationship of recurrence rate and ablation depth, the current consensus recommends a depth of 15 μm in PTK were more suitable (23, 40, 43). Evidence from these studies suggested that a deeper ablation is associated with a lower recurrence rate (23, 43). Recurrence occurred in three eyes (5.77%) in our PTK group, two eyes were relieved after corneal epithelium debridement combined with bandage contact lens application, and one eye received repeat PTK at 1 year postoperatively. Moreover, recurrences of locally trapezoidal design in the first PTK were not included in analysis, hinting higher recurrence rate after PTK. Our findings indicate that PRK combined with concurrent myopic correction yields a favorable prognosis, with a recurrence rate of 6.25%. Notably, the only recurrence occurred in a low-degree myopia patient who underwent PRK with an intraoperative ablation depth of 18 μm. This observation aligns with our speculation that a greater the ablation depth may contribute to a lower recurrence rate, which is in accordance with the results of combined PTK and PRK treatments reported by Zaidman and Hong (29). Meanwhile, we found that the PRK cohort demonstrated milder clinical manifestations during recurrence episodes than the PTK cohort.

The postoperative complications observed in our study included delayed epithelial healing, corneal haze, and corneal nebula. With respect to early complications following excimer laser treatment, delayed epithelial healing was prominent, occurring in two eyes (12.50%) treated with PRK, eight eyes (15.38%) treated with PTK, and one eye (9.09%) treated with TPRK. This was probably due to the extensive ablation zones and impaired epithelial regeneration in patients with RCES. For seven cases with epithelial healing delays exceeding 10 days, manual debridement of the loose epithelium and bandage contact lens replacement were performed. Notably, two of these patients had mild corneal nebula without compromising their final visual acuity. In the majority of studies, epithelialization was completed within 1 week (19, 24, 26), while in some cases, complete epithelialization took up to 35–60 days, which is consistent with our results (9, 22). As the formation and duration of corneal stromal fibrosis are determined by the speed of epithelial basement membrane regeneration, delayed epithelialization exacerbates inflammation in the exposed stroma (44, 45), inducing haze or nebula formation. Proactive intervention involving debridement of loose epithelium is critical when epithelial healing delays exceed 1 week, as this facilitates timely re-epithelialization. Mild corneal haze was found in two eyes after PRK, two eyes after TPRK, and one eye after PTK, suggesting that haze formation is correlated with oblation depth, as described in previous studies (18, 30, 46). Moreover, older age and individual differences may affect corneal epithelialization.

To date, few studies focus on the PRK for RCES accompanied refractive errors. It was firstly mentioned by Bernauer et al. (21), whose effectiveness was only 60% and lack the parameter of oblation zone and the outcomes of visual acuity and refraction. Kremer et al. (30) performed 6 mm PRK in the central cornea and 3-mm overlapping spot PTK at the peripheral area, the effectiveness for RCES reached 100%, but visual acuity decreased in 2 of 16 eyes. Jain et al. (28) also used PRK in the central 6 mm area and PTK in the peripheral area, although the effectiveness for RCES was 92%, partial removal of epithelium and 30 μm ablation depth probably made it hard to predict the visual acuity and refraction. Zaidman et al. (29) performed two-step laser treatment, as PTK followed PRK, with a 6.5 mm ablation zone. They found that the effectiveness for RCES reached 100% and only one eye closed one line of visual acuity. In our cohort, we used a oblation zone range from 7.86 to 9.62 mm (mean 8.92 ± 0.57 mm) with one-step PRK after manually removing full-corneal epithelium. The effectiveness was 93.75%, adjacent to previous studies. Moreover, all eyes acquired a UCVA of 1.0 or better and the mean deviation of the target diopter was −0.25 ± 0.57 D (spherical equivalent) at the final visit, comparable to the results of the non-affected eyes treated with TPRK, which seemed to be more encouraging.

The present study had some limitations. The sample size of PRK and TPRK was relatively small due to less RCES patients accompanied with refractive errors or meeting the eligibility criteria for refractive correction. Meanwhile, the sample size of the groups seemed to be unbalance because of the retrospective design. The large difference of the sample size between the groups made the statistic results less reliable. Therefore, we will design prospective study in the future to validate our findings and explore the risk factors associated with recurrence of RCES furtherly.

## 5 Conclusion

Phototherapeutic keratectomy with extended ablation zone not only treat RCES effectively and safely as PTK, but also yielded excellent visual and refractive outcomes that are comparable to the results of the non-affected eyes treated with TPRK. This approach facilitated simultaneous disease management and refractive correction, ultimately enhancing patient satisfaction.

## Data Availability

The raw data supporting the conclusions of this article will be made available by the authors, without undue reservation.
